# Supplementary material for: Harnessing non-equilibrium forces to optimize work extraction
Source: Nat Commun. 2025 Dec 9;16:11031. doi: 10.1038/s41467-025-67114-8 (PMC12696107; doi:10.1038/s41467-025-67114-8)
Supplement: Supplementary file 1 — Supplementary Information [file 41467_2025_67114_MOESM1_ESM.pdf]

Supplementary information for "Harnessing non-equilibrium forces to optimize work extraction"

Kristian Stølevik Olsen,[1, a)] Rémi Goerlich,[2, 1] Yael Roichman,[2, 3] and Hartmut Löwen[1]
[1)] *Institut für Theoretische Physik II - Weiche Materie, Heinrich-Heine-Universität Düsseldorf, D-40225 Düsseldorf, Germany*
[2)] *Raymond & Beverly Sackler School of Chemistry, Tel Aviv University, Tel Aviv 6997801, Israel*
[3)] *Raymond & Beverly Sackler School of Physics & Astronomy, Tel Aviv University, Tel Aviv 6997801, Israel*

SUPPLEMENTARY NOTE I: EFFECTIVE PARTICLE DYNAMICS IN THE QUASISTATIC LIMIT

The particle dynamics is a result from many competing effects. Indeed, in addition to white noise, the particle experiences arbitrary time-dependent forces as well as a harmonic potential with mobile center. In the optimal protocol however, the trap center also accounts for the forces. This simplifies the effective dynamics of the particle significantly.

The mean particle position within the optimal protocol can be studied from the Euler-Lagrange equation $\gamma\ddot{\mathbf{q}}(t) = \dot{\mathfrak{F}}(t)/2$, which we derived above. Integrating once, we find $\gamma\dot{\mathbf{q}}(t) = \mathfrak{F}(t)/2 + \gamma\boldsymbol{\varphi}(t)$ where $\boldsymbol{\varphi}(t)$ is an additional effective force experienced by the particle on top of $\mathfrak{F}(t)/2$. This is given by Eq. (51) in the main text. In the quasistatic limit, $\gamma\boldsymbol{\varphi}(t) = -\overline{\mathfrak{F}}(t)/2$. The resulting equation for the mean position is simply $\gamma\dot{\mathbf{q}} = \frac{\delta\mathfrak{F}}{2}$. Remarkably, no direct effect of the potential can be seen, and effectively the particle is freely moving under the effect of the force $\frac{\delta\mathfrak{F}}{2}$. Through optimization, the effect of the time-averaged component of the force cancels the effect of the moving harmonic trap. This equation may at first seem paradoxical, since for example in the case of a constant force it states that there is no net movement. Yet, the protocol is designed to transport the particle between two prescribed locations. However, since we work in the quasistatic limit, a finite transport distance performed in infinite time will not contribute to the velocity $\dot{\mathbf{q}}$. Hence the equation $\gamma\dot{\mathbf{q}} = \frac{\delta\mathfrak{F}}{2}$ is naturally interpreted as describing the fast modes of the particle on top of the slow mode that coupled time-averaged force with transport distance.

SUPPLEMENTARY NOTE II: DERIVATION OF EQ. (19)

In the main text, we introduced the M-projection $\pi[p_i]$, defined as

$$\pi[p_i](\mathbf{x}) = \underset{\rho\in\mathcal{B}}{\operatorname{argmin}}\, D_{\mathrm{KL}}(p_i \parallel \rho), \tag{S1}$$

where D_{KL} is the Kullback–Leibler divergence

$$D_{\mathrm{KL}}(p \parallel q) = \int dx p(x) \log \frac{p(x)}{q(x)}. \tag{S2}$$

We here show the steps involved in deriving the equation

$$k_B T\, D_{\mathrm{KL}}(\pi[p_i] \parallel p_{\mathrm{eq}}) = \frac{1}{2} k(\boldsymbol{\lambda}_i - \mathbf{q}_i)^2 \tag{S3}$$

First, we note that when projecting onto a space $\mathcal{B}$ of Boltzmann states compatible with our translational trap manipulations, we can write

$$\rho(x) = \frac{1}{(2\pi\sigma^2)^{d/2}} e^{-\frac{1}{2\sigma^2}(\mathbf{x}-\boldsymbol{\mu})^2} \tag{S4}$$

[a)] *Correspondence: kristian.olsen@hhu.de*

where $\sigma^2 = D/k$ is the equilibrium variance and d is the dimension. In this case, we have

$$D_{\mathrm{KL}}(p_i \parallel \rho) = -S[p_i] - \int d\mathbf{x} p_i(\mathbf{x}) \log\left[\frac{1}{(2\pi\sigma^2)^{d/2}} e^{-\frac{1}{2\sigma^2}(\mathbf{x}-\boldsymbol{\mu})^2}\right] \tag{S5}$$

where $S[p_i]$ is the Shannon-Gibbs entropy. Since we want to minimize this divergence with respect to ρ, the only free parameter to vary is the location $\boldsymbol{\mu}$. Hence, we only need the terms dependent on this, i.e.,

$$D_{\mathrm{KL}}(p_i \parallel \rho) = \int d\mathbf{x} p_i(\mathbf{x}) \frac{1}{2\sigma^2}(\mathbf{x}-\boldsymbol{\mu})^2 + ... \sim \langle \mathbf{x}^2 \rangle_i - 2\boldsymbol{\mu}\mathbf{q}_i + \boldsymbol{\mu}^2 \tag{S6}$$

where the subscript $i$denotes expectation values with respect to $p_i(\mathbf{x})$. Solving $\partial_{\boldsymbol{\mu}} D_{\mathrm{KL}}(p_i \parallel \rho) = 0$ immediately gives $\boldsymbol{\mu} = \mathbf{q}_i$, and hence the projected initial state is simply

$$\pi[p_i](\mathbf{x}) = \frac{1}{(2\pi\sigma^2)^{d/2}} e^{-\frac{1}{2\sigma^2}(\mathbf{x}-\mathbf{q}_i)^2} \tag{S7}$$

We can now calculate

$$k_B T\, D_{\mathrm{KL}}(\pi[p_i] \parallel p_{\mathrm{eq}}) = k_B T \int dx \pi[p_i](\mathbf{x}) \log \frac{\pi[p_i](\mathbf{x})}{p_{\mathrm{eq}}(\mathbf{x})} = -k_B T S[\pi[p_i]] + \int d\mathbf{x}\, \pi[p_i](\mathbf{x}) V[\mathbf{x}, \boldsymbol{\lambda}_i] + \frac{d}{2} k_B T \log(2\pi\sigma^2) \tag{S8}$$

where we used that the instantaneous equilibrium state at the initial time is a Boltzmann state centered at $\boldsymbol{\lambda}_i$. Using the well-established Shannon entropy for a Gaussian to calculate $S[\pi[p_i]]$, and performing the Gaussian integral $\int d\mathbf{x}\, \pi[p_i](\mathbf{x}) V[\mathbf{x}, \boldsymbol{\lambda}_i]$ with a harmonic trap $V[\mathbf{x}, \boldsymbol{\lambda}_i] = \frac{k}{2}(\mathbf{x} - \boldsymbol{\lambda}_i)^2$ one arrives at

$$k_B T\, D_{\mathrm{KL}}(\pi[p_i] \parallel p_{\mathrm{eq}}) = \frac{k}{2}(\boldsymbol{\lambda}_i - \mathbf{q}_i)^2 \tag{S9}$$

as we wanted to show.

SUPPLEMENTARY NOTE III: TIME-INTEGRATED MOMENTS OF THE ACTIVE FORCES

In the main text we considered an active particle which experiences a mean self-propulsion force

$$\boldsymbol{\mathfrak{F}}(t|\phi_0) = \langle f_0 \hat{\mathbf{n}}(t) | \phi_0 \rangle = f_0 \begin{bmatrix} \cos(\phi_0) e^{-\frac{1}{2}\sigma_\phi^2(t)} \\ \sin(\phi_0) e^{-\frac{1}{2}\sigma_\phi^2(t)} \end{bmatrix} \tag{S10}$$

In order to calculate optimal protocols and the associated work, we need the first two time-integrated moments

$$\overline{\mathfrak{F}_\alpha^n} = \frac{1}{t} \int_0^t dt \mathfrak{F}_\alpha^n(t)\ ,\ n = 1, 2, \tag{S11}$$

where $\alpha \in \{x, y\}$ determines the spatial directions. Since the time-dependence only comes from the exponential factor $e^{-\frac{1}{2}\sigma_\phi^2(t)}$ with $\sigma_\phi^2(t) = 2D_r(t - \tau_R(1 - e^{-t/\tau_R}))$ we only consider this integral. We have to perform the integral

$$I_n = \int_0^t dt \left(e^{-D_r(t-\tau_R(1-e^{-t/\tau_R}))} \right)^n \tag{S12}$$

We start with I_1, for which we perform the substitution $u \equiv e^{-t/\tau_R}$, transforming the integral into

$$I_1 = \tau_R \int_{e^{-t/\tau_R}}^1 du\, u^{D_r\tau_R - 1} e^{D_r\tau_R(1-u)} \tag{S13}$$

This can be performed exactly using the incomplete Gamma function, resulting in

$$I_1 = \frac{\tau_R e^{-D_r\tau_R}}{(D_r\tau_R)^{D_r\tau_R}} \left[\Gamma\left(D_r\tau_R \middle| D_r\tau_R e^{-t/\tau_R} \right) - \Gamma\left(D_r\tau_R \middle| D_r\tau_R \right) \right] \tag{S14}$$

Since the integrand is purely exponential, we also immediately see that for the second moment, it sufficed to observe that $I_2(D_r) = I_1(2D_r)$.

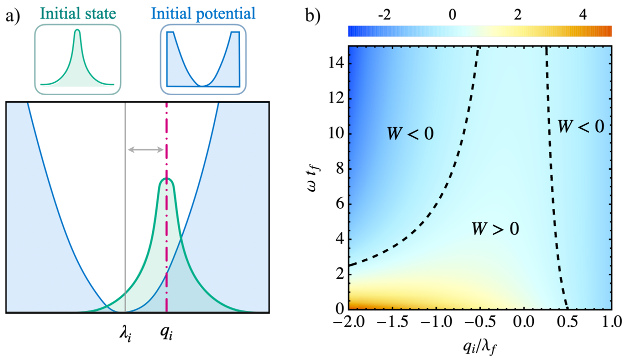

Supplementary Figure S1. a) Sketch of the initial state, which is assumed to be a non-Boltzmann state with mean $q_i \neq \lambda_i$. b) Work, measured in units of $\frac{k}{2}\lambda_f^2$, as a function of protocol duration and initial mean position.

SUPPLEMENTARY NOTE IV: BASELINE EXAMPLE: NO FORCES

As a baseline example, we consider the case of no forces. At the initial time $t = 0$ we assume that the particle has been initialized away from the Boltzmann state associated with the trap, so that $q_i \neq \lambda_i$. See Fig.(S1a) for a sketch. Since there are no external driving forces in this case, the optimal protocol is simply $\lambda^* = \lambda_{\rm eq}$. The effect of this initialization is seen both in the optimal protocol and in the associated work. The protocol has discontinuous jumps both at its beginning and end, a common feature of such protocols[1]. At the beginning the jump is $\Delta\lambda_i = \lambda^*(0) - \lambda_i$, and at the end $\Delta\lambda_f = \lambda_f - \lambda^*(t_{\rm f})$. From the main results, we have

$$\Delta\lambda_i = \Delta\lambda_f + q_i - \lambda_i \tag{S15}$$

with $\Delta\lambda_f = (\lambda_f - q_i)/(2 + \omega t_{\rm f})$. Hence, jumps are asymmetric ($\Delta\lambda_i \neq \Delta\lambda_f$) if the particle is initialized away from the trap center. Furthermore, while the discontinuous jumps typically vanish in the quasistatic limit, here the non-equilibrium initial state leads to an initial jump of size $q_i - \lambda_i$ even in the quasistatic limit. This initial jump mimics instantaneous equilibration protocols where the protocol instantaneously changes to conform to the initial state[2,3].

The initial non-equilibrium state also has implications for finite-time protocols. The thermodynamic work takes the form

$$\mathcal{W} = k\frac{(\lambda_f - q_i)^2}{2 + \omega t_{\rm f}} - \frac{1}{2}k(\lambda_i - q_i)^2. \tag{S16}$$

The phase space over which positive and negative regions can be identified is shown in Fig.(S1b). The zero-work lines, at fixed $t_{\rm f}$, can be predicted exactly and are given by the two initial positions

$$\frac{q_i^\pm}{\lambda_f} = \frac{1}{1 \pm \sqrt{1 + \frac{\omega t_{\rm f}}{2}}} \tag{S17}$$

While in the quasi-static limit, any initial deviation from the trap center results in work extraction, for finite-time protocols a sufficiently large displacement is needed.

[1]T. Schmiedl and U. Seifert, "Optimal finite-time processes in stochastic thermodynamics," Physical review letters **98**, 108301 (2007).
[2]M. Esposito and C. Van den Broeck, "Second law and landauer principle far from equilibrium," Europhysics Letters **95**, 40004 (2011).
[3]J. M. Parrondo, J. M. Horowitz, and T. Sagawa, "Thermodynamics of information," Nature physics **11**, 131–139 (2015).
